# Development of a Global Functionality Index Integrating Physical Performance and Wearable-Derived Outdoor Walking Metrics in Older Adults

**DOI:** 10.3390/s26154722

**Published:** 2026-07-25

**Authors:** José Carlos Cabrera Linares, Juan Antonio Párraga Montilla, Pedro Ángel Latorre Román

**Affiliations:** Department of Musical, Plastic and Corporal Expression, University of Jaén, 23071 Jaén, Spain; jparraga@ujaen.es (J.A.P.M.); platorre@ujaen.es (P.Á.L.R.)

**Keywords:** functional capacity, older adults, wearable devices, gait analysis, physical performance

## Abstract

Objective: To develop and internally evaluate a Global Functionality Index (GFI) in older adults and to examine the combined influence of age, sex, and body mass index (BMI) on functional capacity. Methods: A total of 110 older adults (70.89 ± 5.93 years) completed a physical performance battery including gait, muscle strength, and endurance assessments. Wearable-derived outdoor walking speed was measured using a Garmin Forerunner 635 and HRM-Pro band. Exploratory principal component analysis, k-means clustering, and multivariable linear and logistic regression were performed. Results: Women had lower GFI values than men (*p* < 0.001). Although overall GFI differences by age group were not significant, women aged 70 years and older showed significantly lower GFI values than women younger than 70 years (*p* = 0.018). In the multivariable linear models, age and sex were the main independent correlates of GFI (both *p* < 0.001), whereas BMI showed a borderline, non-significant association (*p* = 0.066). In logistic regression, age was associated with membership in the lowest GFI quartile (*p* = 0.008), while BMI showed a borderline, non-significant association (*p* = 0.065). Cluster analysis identified three distinct functional profiles. Conclusions: The GFI represents a derived multidimensional index of functional capacity that integrates laboratory-based performance tests and wearable-derived outdoor performance metrics. These findings support the preliminary interpretation of the GFI as a sample-dependent research index derived within this cohort; they do not establish external or predictive validity.

## 1. Introduction

Population aging is a major public-health achievement, but it also creates substantial challenges for health and social-care systems, social structures, and the overall quality of life of older adults. As global life expectancy continues to increase, the gerontological focus has shifted from merely prolonging survival to promoting healthy aging. This paradigm is defined not only by longevity but by the preservation of functional independence throughout the lifespan [1]. In this context, the World Health Organization (WHO) has introduced the “Intrinsic Capacity” model, a construct that advocates for the holistic measurement of an individual’s physical and mental reserves to prevent the cascade of dependence [2]. Within this framework, functional capacity emerges as a central element; it is not simply a reflection of musculoskeletal health, but an integrative indicator of the individual’s ability to perform daily activities and maintain autonomy [3].

Functional capacity is a complex, multidimensional concept that integrates physical, physiological, and cognitive domains. Among these, physical performance has been identified as a key determinant of independence and a strong predictor of adverse outcomes, including disability, institutionalization, and mortality [4]. Measures such as gait speed, handgrip strength (HS), and performance-based functional tests have been widely used to assess these domains. Gait speed, in particular, has been extensively studied as a robust indicator of overall health status, earning the title of the “vital sign” in geriatric populations due to its strong association with survival and functional decline [5,6]. Similarly, HS is recognized as an indispensable proxy for systemic vitality and a primary diagnostic criterion for sarcopenia [6,7]. Furthermore, dynamic functional assessments, such as the sit-to-stand test and the 6 min walk test (6MWT), provide essential insights into lower-limb power, endurance, and mobility, capturing integrative aspects of functional performance that extend beyond isolated measures of maximal strength [8,9].

Despite the widespread clinical use of these individual measures, a primary challenge lies in their fragmentation. Each test captures a specific domain of physical performance, but no single measure fully represents overall functional status [10]. To address this limitation, several composite indices have been developed to capture broader and more integrative aspects of functional capacity, including the Short Physical Performance Battery [11], the frailty phenotype [12], and multidimensional approaches such as the Frailty Index and functional fitness batteries, which attempt to incorporate multiple domains of physical performance and health status [13,14]. While these tools represent a step forward, they frequently exhibit “ceiling effects” (i.e., when high-functioning individuals easily achieve maximum scores, thereby masking early or subtle declines in physical capacity), limiting their sensitivity to detect subtle performance reductions in robust or pre-frail older adults [15,16]. Moreover, they often focus on a restricted set of variables, sometimes overlooking the continuous integration of upper-body strength, dynamic power, and cardiorespiratory endurance [17,18].

Compounding the effects of age and sex, body composition also plays an important role. Aging drives a detrimental tissue redistribution, depleting lean mass while expanding visceral adiposity. This excess fat imposes an increased biomechanical load that hinders mobility and triggers metabolic disturbances [19]. Clinically, the most adverse phenotype emerges from the synergistic convergence of high adiposity and impaired muscle function, formally defined as “sarcopenic obesity” [20]. Beyond mechanical constraints, this condition fosters chronic low-grade inflammation and lipotoxicity, which blunt neuromuscular activation. Consequently, sarcopenic obesity substantially increases the risk of severe functional decline and disability compared to either condition in isolation [21,22].

Given the multidimensional and heterogeneous nature of functional decline, there is a clear need for comprehensive, standardized, and continuous indices capable of capturing functional performance across multiple domains. While traditional performance-based laboratory tests offer valuable, standardized snapshots of physical capacity, they often fail to reflect an individual’s true behavior outside the clinical setting [23,24]. In recent years, advances in wearable technology have addressed this gap by enabling the objective assessment of physical activity and spatiotemporal movement patterns in free-living conditions, providing ecologically valid insights into real-world functional behavior [25]. In particular, consumer-grade wearable devices equipped with GPS and inertial sensors may provide objective estimates of walking speed, distance, and activity patterns in outdoor environments [26]. Despite these technological advances, effectively integrating large volumes of continuous real-world data with discrete laboratory assessments into a single, meaningful, and interpretable metric remains a significant methodological challenge [27].

In this context, the Global Functionality Index (GFI) was conceived to address the lack of a continuous index integrating complementary laboratory-based functional domains with wearable-derived outdoor gait metrics. Whereas isolated gait speed captures a single mobility domain, the GFI integrates multiple functional domains; in contrast to the Frailty Index, it is based exclusively on objective physical performance; and compared with the SPPB and traditional functional fitness batteries, it preserves continuous information on upper- and lower-body performance, endurance, gait efficiency, and wearable-derived outdoor walking speed. The wearable-derived component adds information on outdoor locomotor performance beyond strictly laboratory-based assessments. However, the present study does not establish superior diagnostic, prognostic, or clinical value compared with established functional assessment tools. This approach aims to reduce the fragmentation of single-domain assessments by combining controlled performance testing with standardized outdoor gait data. Therefore, the objective of this study was to develop and internally evaluate a GFI in older adults and to examine the combined influence of age, sex, and anthropometric indicators on functional capacity.

## 2. Materials and Methods

### 2.1. Participants

This cross-sectional study included a total of 110 older adults (76 women; mean age = 70.89 ± 5.93 years; body mass index [BMI] = 27.12 ± 3.92 kg/m^2^). An a priori power analysis was conducted using G*Power software (version 3.1.9.7) to determine the appropriate sample size. Assuming a two-tailed independent samples *t*-test to detect a moderate effect size (Cohen’s d = 0.60) in functional performance between participants aged < 70 and ≥70 years, with a significance level of α = 0.05 and a statistical power of 1 − β = 0.80, a minimum sample size of 90 participants was required. Additional multivariable linear regression, logistic regression, and cluster analyses were considered exploratory and interpreted accordingly.

Participants were recruited from several senior associations located in southern Andalusia, Spain. The sample was divided into two age-based groups: Group 1, comprising individuals younger than 70 years, and Group 2, including those aged 70 years and older. This specific threshold was selected based on established literature identifying 70 years as a critical inflection point for significant declines in physical performance and spontaneous gait speed [28,29].

The inclusion criteria were as follows: (a) age of 60 years or older; (b) independent ambulation ability; (c) absence of any medical condition requiring daily medication that could affect gait performance; and (d) no prior diagnosis of disorders associated with an increased risk of falls. All participants provided written informed consent prior to participation. The study was conducted in accordance with the ethical principles outlined in the Declaration of Helsinki (2013) and received approval from the Ethics Committee of the University of Jaén (Reference code: OCT.20/7.PRY. 26 October 2020).

### 2.2. Materials and Testing Procedures

Body mass was measured using a Seca 899 scale (Seca GmbH & Co. KG, Hamburg, Germany), while height was assessed with a stadiometer (Seca 222, Seca GmbH & Co. KG, Hamburg, Germany). Body mass index (BMI) was then determined as body mass in kilograms divided by the square of height in meters. Blood pressure was measured using an automated oscillometric device (HEM-907XL; OMRON Healthcare Co., Ltd., Kyoto, Japan).

#### 2.2.1. Clinical, Cognitive, Health-Related Quality of Life, and Lifestyle Assessment

Clinical, cognitive, health-related quality of life, and lifestyle variables were assessed to characterize the sample and to examine their associations with the derived GFI as external variables; they were not included as components of the index. Sociodemographic and contextual characteristics are presented in Supplementary Table S1. Health-related quality of life was assessed using the 12-item Short Form Health Survey (SF-12), which provides two summary measures: the Physical Component Summary (PCS) and the Mental Component Summary (MCS) [30]. Higher scores indicated better quality of life. In addition, fall history was assessed by asking participants to report the number of falls experienced during the previous 3 years. A fall was defined as an event in which the individual unintentionally came to rest on the floor or another lower surface, such as a bed or chair.

Global cognitive performance was assessed using the Test Your Memory (TYM), which is a brief self-administered cognitive screening instrument designed to be completed in approximately 5 min. The total score ranges from 0 to 50, with higher scores indicating better cognitive performance. The TYM comprises 10 tasks covering 11 cognitive domains: orientation, sentence copying, semantic knowledge/retrograde memory, calculation, phonemic verbal fluency, abstraction, naming, visuospatial abilities, anterograde memory, and executive function, defined as the ability to complete the test independently [31].

Comorbidity burden was assessed using the Spanish version of the Charlson Comorbidity Index (CCI) [32]. The CCI is a weighted comorbidity measure designed to estimate the prognostic impact of chronic coexisting conditions on 1-year mortality. It includes 17 diagnostic categories, each weighted 1, 2, 3, or 6 points according to prognostic relevance. The total score was calculated by summing the weights of all recorded comorbidities, with higher scores indicating greater comorbidity burden and poorer prognosis.

#### 2.2.2. Physical Performance

Physical activity level was assessed using a brief version of the International Physical Activity Questionnaire (IPAQ), Spanish adaptation [33]. This instrument evaluated engagement in moderate-to-vigorous physical activity as well as sedentary behavior during the previous seven days.

The six GFI components were selected a priori to capture complementary domains of functional capacity commonly assessed in older adults. All selected measures are based on functional tests frequently used in this population, and the supporting references for each test are provided in the corresponding test description. This selection was not based on a formal systematic review or consensus process, but on established gerontological assessment domains: lower-limb functional strength (10-STST), maximal gait performance (10 m gait speed), upper-body neuromuscular capacity (HS), cardiorespiratory endurance (6MWT), gait efficiency (walk ratio), and outdoor walking performance (wearable-derived walking speed).

Walk ratio, calculated as step length divided by cadence, was included as an indicator of gait efficiency and spatiotemporal gait organization, because it reflects how step length and cadence are coordinated during walking rather than considering either parameter in isolation. Therefore, step length and cadence were not included as independent components of the GFI, but were integrated through walk ratio to reduce redundancy and represent gait efficiency.

Isometric HS was measured using a digital dynamometer (T.K.K. 5101 Grip-D, Takei Scientific Instruments Co., Ltd., Tokyo, Japan). Each participant performed two maximal trials with each hand, and the best trial was used for subsequent analysis. The test was conducted in a standing position, with the arm fully extended alongside the body and not in contact with the trunk. A minimum rest interval of 60 s was allowed between trials [7].

Gait speed (GS) was assessed using two double-light timing gates (WITTY; Microgate Srl, Bolzano, Italy; accuracy: 0.001 s), positioned at the beginning and end of a 10 m walkway. Participants were instructed to walk the distance as quickly as possible without running. The fastest of two trials was retained for analysis. No explicit start signal was provided, allowing participants to initiate movement at their own discretion.

Lower-limb functional performance was assessed using the 10-repetition sit-to-stand test (10-STST). Participants were instructed to stand up and sit down ten times as quickly as possible from a standard chair without armrests, with their arms crossed over the chest. The total time required to complete the ten repetitions was recorded using a stopwatch, starting at the command “go” and ending upon completion of the final repetition. Shorter times indicated better performance [34].

Cardiorespiratory fitness was evaluated using the 6 min walk test (6MWT). Participants were instructed to walk as far as possible, without running, around a 50 m rectangular circuit for 6 min. The total distance covered was recorded in meters [13]. A greater distance indicated better cardiorespiratory fitness.

Wearable-derived outdoor gait metrics were recorded using a Garmin Forerunner 635 smartwatch paired with a heart rate monitor (HRM-Pro; Garmin Ltd, Olathe, KS, USA). The smartwatch was worn on the left wrist and the HRM-Pro band on the chest, following the manufacturer’s instructions. Consumer-grade Garmin devices have shown acceptable accuracy for recording distance- and speed-related metrics in outdoor or running-based contexts [26,35]. In the present study, all participants completed the same standardized 50 min outdoor walking task at a self-selected pace. This single assessment was intended to provide a standardized sample of outdoor locomotor performance and should not be interpreted as representative of habitual free-living gait. Therefore, these variables should be interpreted as wearable-derived gait metrics obtained under semi-controlled outdoor conditions, rather than as habitual daily life monitoring. Average walking speed was extracted as the wearable-derived speed component of the GFI, while step length and cadence were used to calculate the walk ratio. No additional signal filtering or post-processing was applied beyond the device-derived output. Device-specific test–retest reliability, measurement reproducibility, and GPS signal-quality indicators were not assessed in the present study.

### 2.3. Procedure

**Figure 1:**
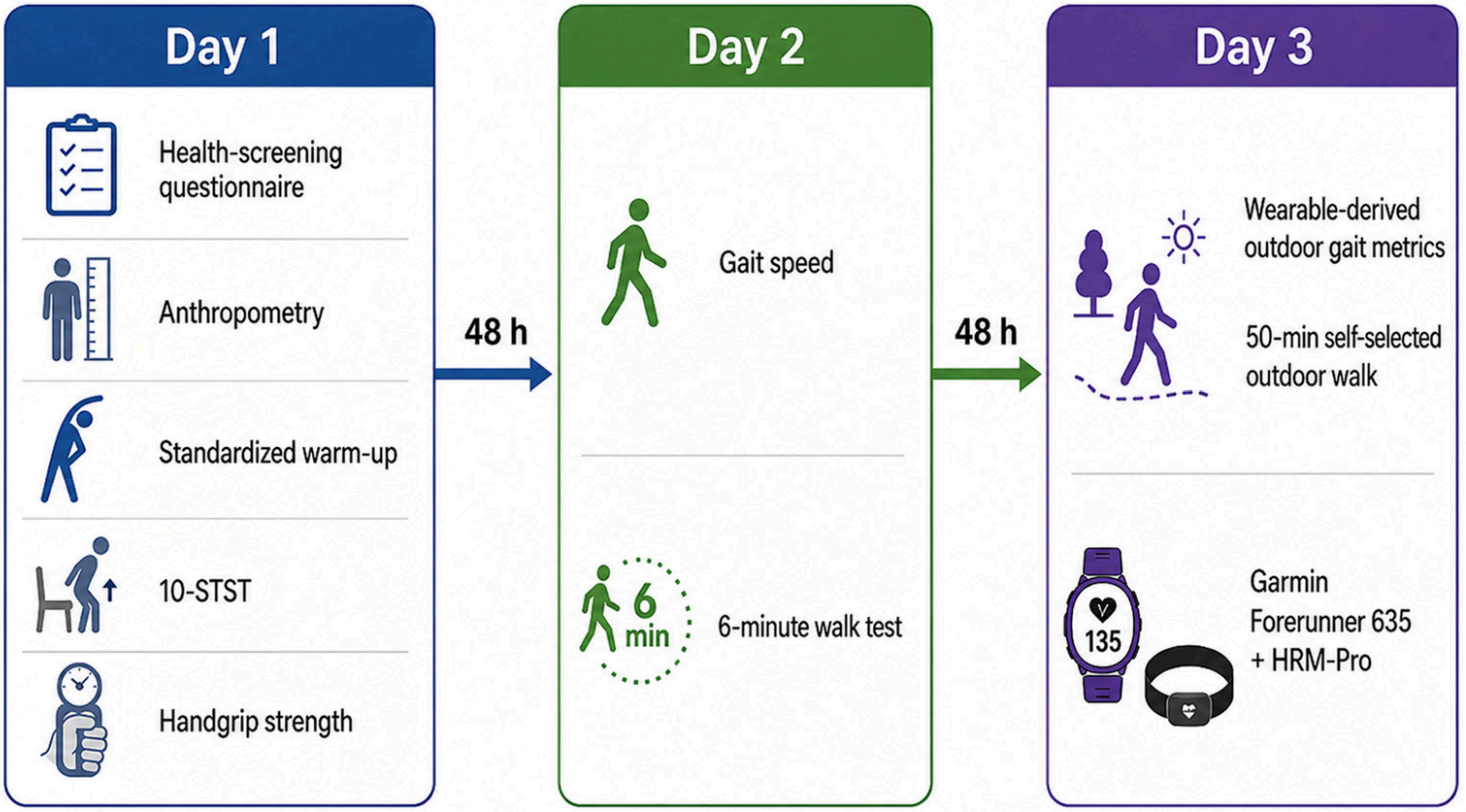
Timeline of the assessment protocol across the three testing sessions.

The assessment protocol was implemented over three sessions separated by 48 h. Participants were instructed to abstain from vigorous physical activity and alcohol intake for at least 24 h prior to each session. On the first day, upon arrival, participants completed a health-screening questionnaire, followed by anthropometric measurements. A standardized warm-up, consisting of 10 min of low-intensity aerobic exercise and dynamic mobility, was performed before the physical assessments. Subsequently, the 10-STST and HS were conducted. On the second day, GS and the 6MWT were evaluated. On the third day, wearable-derived outdoor gait metrics were assessed. When appropriate, participants completed a familiarization trial prior to data collection. The order of assessments was standardized as follows: Day 1: (1) health questionnaire, (2) anthropometry, (3) 10-STST, and (4) HS; Day 2: GS and 6MWT; Day 3: wearable-derived outdoor gait metrics (Figure 1). To control for potential order effects, the initial test within each session was randomly assigned.

### 2.4. Statistical Analysis

All statistical analyses were conducted using Python 3.10.12 (pandas, scipy, scikit-learn, and statsmodels libraries). Statistical significance was set at *p* < 0.05. The GFI was constructed by integrating the six physical performance variables described above: 10-STST, gait speed over 10 m, HS, 6MWT, walk ratio, and wearable-derived outdoor walking speed. Fall history was not included in the GFI because it represents a retrospective clinical outcome rather than a standardized physical performance metric. Higher values indicated better performance for gait speed, HS, 6MWT, walk ratio, and wearable-derived outdoor walking speed. Because lower values indicate better performance in the 10-STST, this variable was inverted before standardization. Each component was standardized using the pooled sample mean and standard deviation according to the following formula: z_i_ = (x_i_ − mean)/SD. The GFI raw score was calculated as the unweighted mean of the six standardized components: GFIraw = (z10-STST + z10m speed + zHS + z6MWT + zwalk ratio + zoutdoor speed)/6. The resulting score was then transformed to a 0–100 scale using min–max rescaling: GFI = [(GFIraw − GFIraw min)/(GFIraw max − GFIraw min)] × 100. Higher GFI values indicate better global functional capacity. Participants who were initially assessed but did not complete all testing sessions were not included in the final analytical sample. Among the 110 participants included in the study, complete data were available for all six GFI components; therefore, no participant was excluded from the GFI calculation due to missing component data. A sensitivity analysis was also performed by recalculating the GFI using sex-stratified z-scores for the component variables.

In addition, equal weighting was selected to preserve transparency and reproducibility because all six variables were standardized before aggregation and represented complementary functional domains selected a priori. PCA-derived weights were not used because the PCA was exploratory and performed on the same variables used to derive the index, which could have overfitted the scoring procedure to the present sample. Clinically informed weighting was not applied because no validated weighting scheme exists for this specific combination of laboratory-based and wearable-derived outdoor functional variables. As a sensitivity analysis, an alternative PCA-weighted GFI was calculated using the retained principal components weighted by their explained variance and was compared with the equal-weighted GFI. To describe the internal structure of the selected functional variables, a principal component analysis (PCA) was performed on the standardized variables. This analysis was considered exploratory and was not interpreted as external validation of the GFI. The adequacy of the dimensional structure was assessed based on explained variance and variable loadings.

Sex differences in GFI and functional variables were analyzed using independent samples *t*-tests. Participants were stratified into two age groups (<70 years and ≥70 years), and between-group differences were assessed using independent samples *t*-tests. Additionally, a two-way analysis (sex × age group) was conducted to evaluate interaction effects on GFI.

Pearson correlation analyses were performed to examine the association between GFI and age. Furthermore, partial Pearson correlations adjusted for age and sex were conducted to evaluate the independent associations between GFI and anthropometric (BMI, waist circumference), clinical (blood pressure), cognitive (TYM), and health-related quality of life variables (SF-12), as well as falls and comorbidity (CCI). In addition, a multivariable linear regression model was constructed to examine independent associations with GFI. Only variables not included in the index were considered as explanatory variables, including age, sex, BMI, systolic blood pressure, SF-12 physical and mental scores, cognitive performance (TYM), number of falls, CCI, and physical activity level (IPAQ). This distinction was made to avoid circular interpretation between variables used to construct the GFI and variables used to examine external associations with the index. Linear regression assumptions were examined using residual normality, homoscedasticity, variance inflation factors, leverage values, and Cook’s distance.

To explore sample-specific functional profiles, k-means cluster solutions were examined using the standardized functional variables included in the GFI; this analysis was descriptive and was not considered evidence of index validation. The three-cluster solution was retained because it provided an interpretable exploratory stratification into low, moderate, and high functional profiles; no formal cluster validation metric was used. 10-STST values were inverted prior to clustering. Clusters were ordered according to mean GFI values and classified as low (Cluster 0), moderate (Cluster 1), and high functionality (Cluster 2). Differences across clusters were analyzed using one-way ANOVA, and effect sizes were calculated using eta-squared (η^2^). Finally, a binary logistic regression analysis was conducted to examine factors associated with low functional capacity. For the exploratory logistic regression, low GFI was defined as values below the 25th percentile of the sample distribution to identify the lowest-functioning subgroup within this cohort. This threshold was sample-specific and was not intended to represent a diagnostic or clinically validated cut-off. Age and BMI were included as explanatory variables based on prior evidence and correlation analyses.

## 3. Results

### 3.1. Sociodemographic Data of the Participants

A total of 110 older adults were included in the study. Sociodemographic characteristics stratified by sex and age group are presented in Supplementary Table S1. Most participants had a medium to high educational level (41.2% medium and 33.8% high). The majority were retired (88.8%), and 52.5% reported having had a previous occupation. Regarding marital status, most participants were married (63.8%), followed by widowed (21.2%), divorced (10.0%), and single (5.0%). A high proportion of participants reported living alone (65.0%), and almost all resided in urban areas (97.5%). In terms of socioeconomic status, most individuals reported a medium income level (50.0%), followed by medium–low (36.2%). Regarding lifestyle factors, 57.5% were non-smokers, while alcohol consumption was mainly moderate (52.5%) or absent (46.2%).

### 3.2. Functional Performance

The combined analysis by sex and age group revealed distinct and complementary patterns in functional performance and clinical characteristics (Table 1). Men consistently exhibited higher values than women in several key physical parameters, including HS, gait speed (10 m), step length, walk ratio (*p* < 0.001), and wearable-derived outdoor walking speed (*p* = 0.004). In contrast, women showed poorer performance in the 10-STST (*p* = 0.014). Men also presented higher systolic (*p* = 0.004) and diastolic blood pressure (*p* = 0.014), whereas no sex differences were observed in BMI, quality of life (SF-12), cognitive performance, or falls.

When stratified by age, participants aged ≥ 70 years showed lower functional performance compared to those < 70 years, particularly in gait-related variables such as 10 m walking speed (d = 0.43), step length (d = 0.39), and wearable-derived outdoor walking speed (d = 0.41), whereas CCI values differed between age groups, although not in the expected age-related direction (d = 0.52). However, the magnitude of these age-related differences was consistently smaller than those observed between sexes.

### 3.3. Construction and Internal Structure of the Global Functionality Index

**Figure 2:**
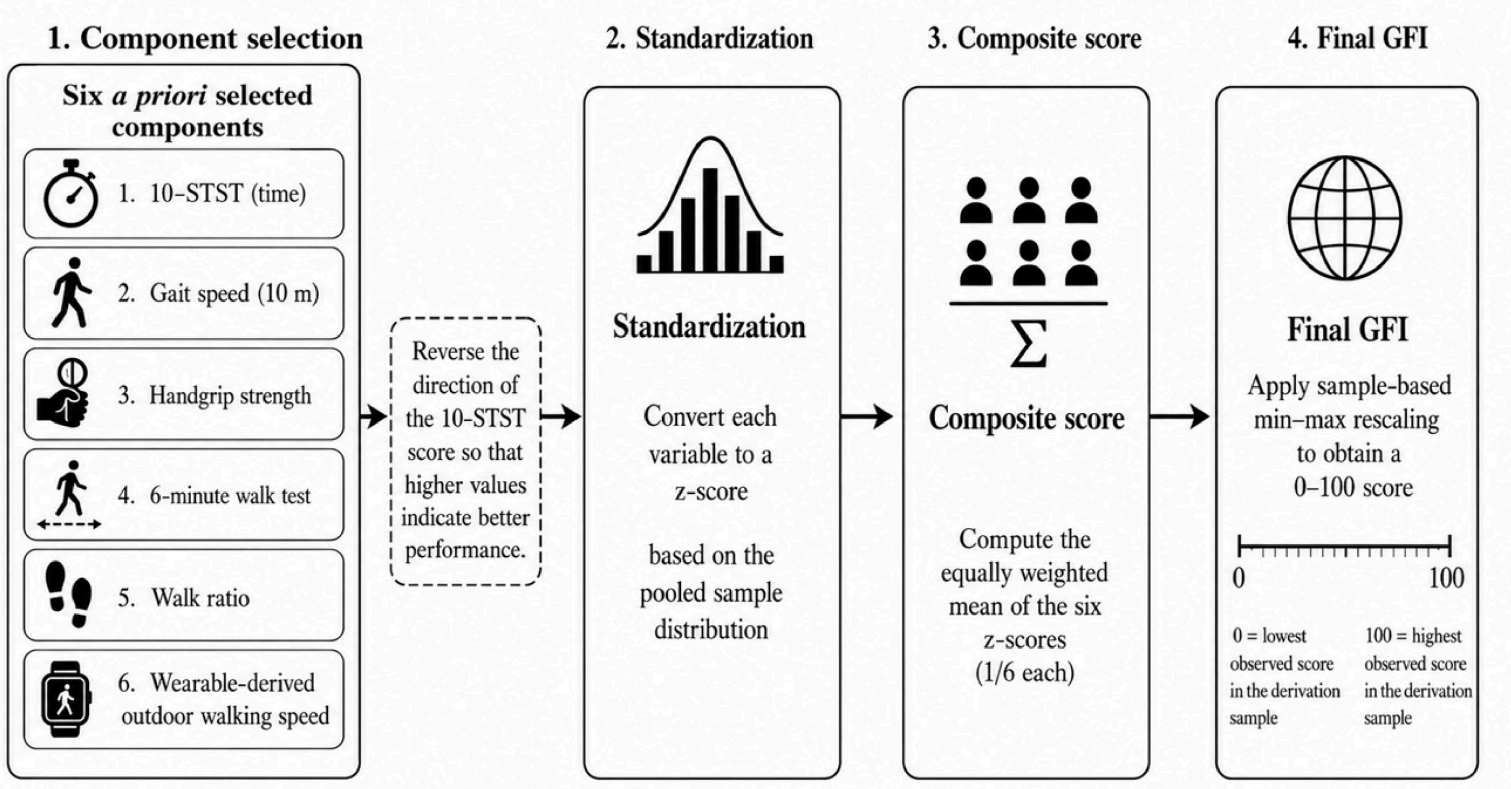
Construction workflow of the Global Functionality Index.

The workflow used to construct the GFI, including component selection, directional alignment, pooled-sample standardization, equal weighting, and sample-based min–max rescaling, is presented in Figure 2.

**Figure 3:**
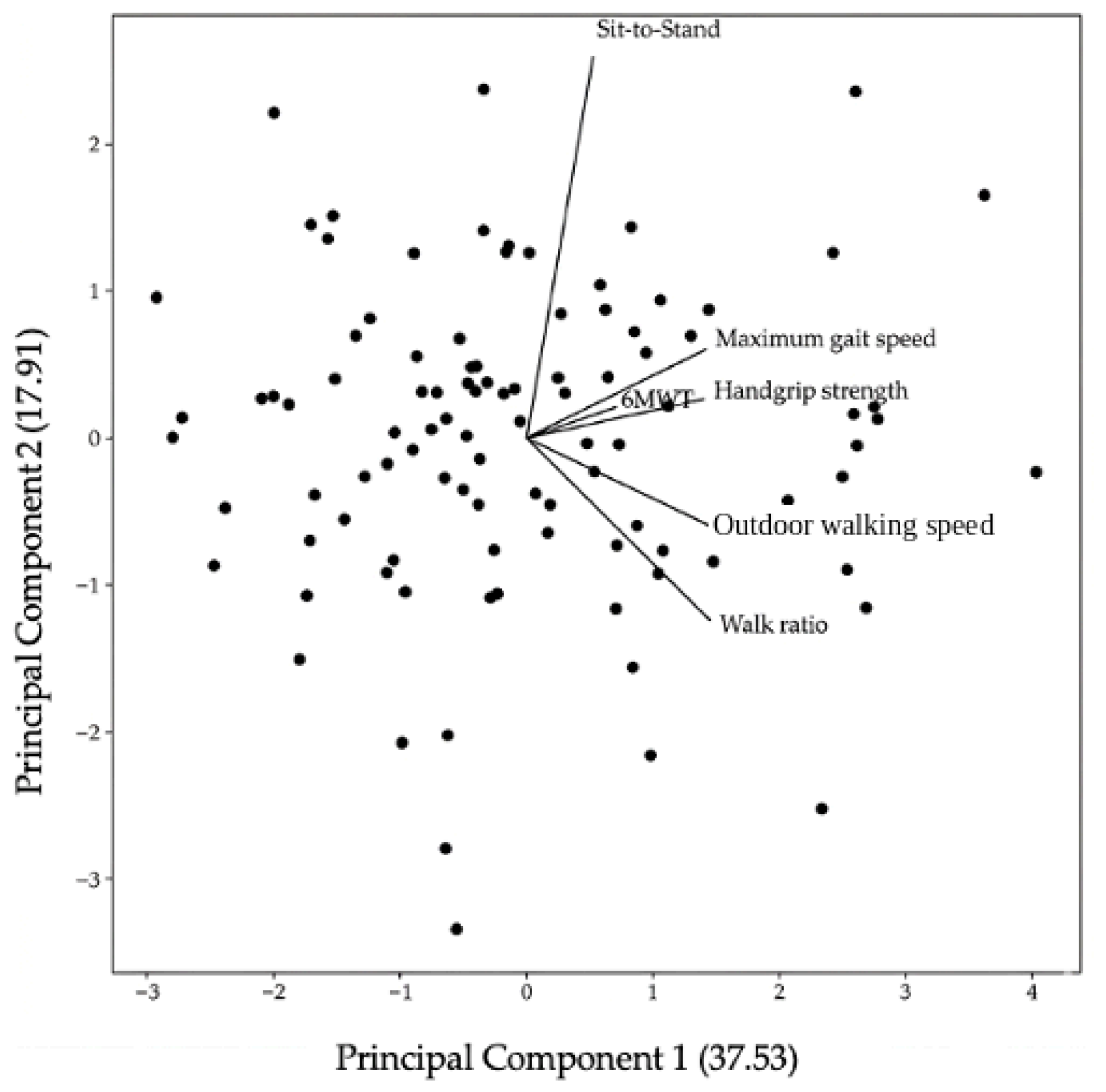
Principal component analysis biplot of the six standardized physical performance variables included in the Global Functionality Index.

After constructing the index, PCA was performed on the six standardized component variables to explore their internal structure (Figure 3). The first two principal components had eigenvalues >1 and together explained 55.44% of the total variance (PC1: eigenvalue = 2.252, 37.53%; PC2: eigenvalue = 1.074, 17.91%). This indicates a moderate internal structure, with a substantial proportion of variance remaining outside the first two components. PC3 had an eigenvalue below 1 (0.948), and the scree plot suggested a modest inflection after the second component. PC1 showed higher loadings for wearable-derived outdoor walking speed, walk ratio, 10 m gait speed, and HS, suggesting a general locomotor-performance dimension. PC2 was mainly driven by the inverted 10-STST, reflecting a lower-limb functional strength dimension. Full PCA results, including eigenvalues, explained variance, component loadings, and communalities, are provided in Supplementary Table S2. Sex-specific intercorrelations among the six GFI component variables are shown in Supplementary Figure S1. The equal-weighted GFI was very strongly correlated with the PCA-weighted alternative formulation (r = 0.982, *p* < 0.001), and the main sex- and age-related patterns were unchanged.

### 3.4. Age- and Sex-Related Patterns in Global Functionality and Explanatory Model

**Figure 4:**
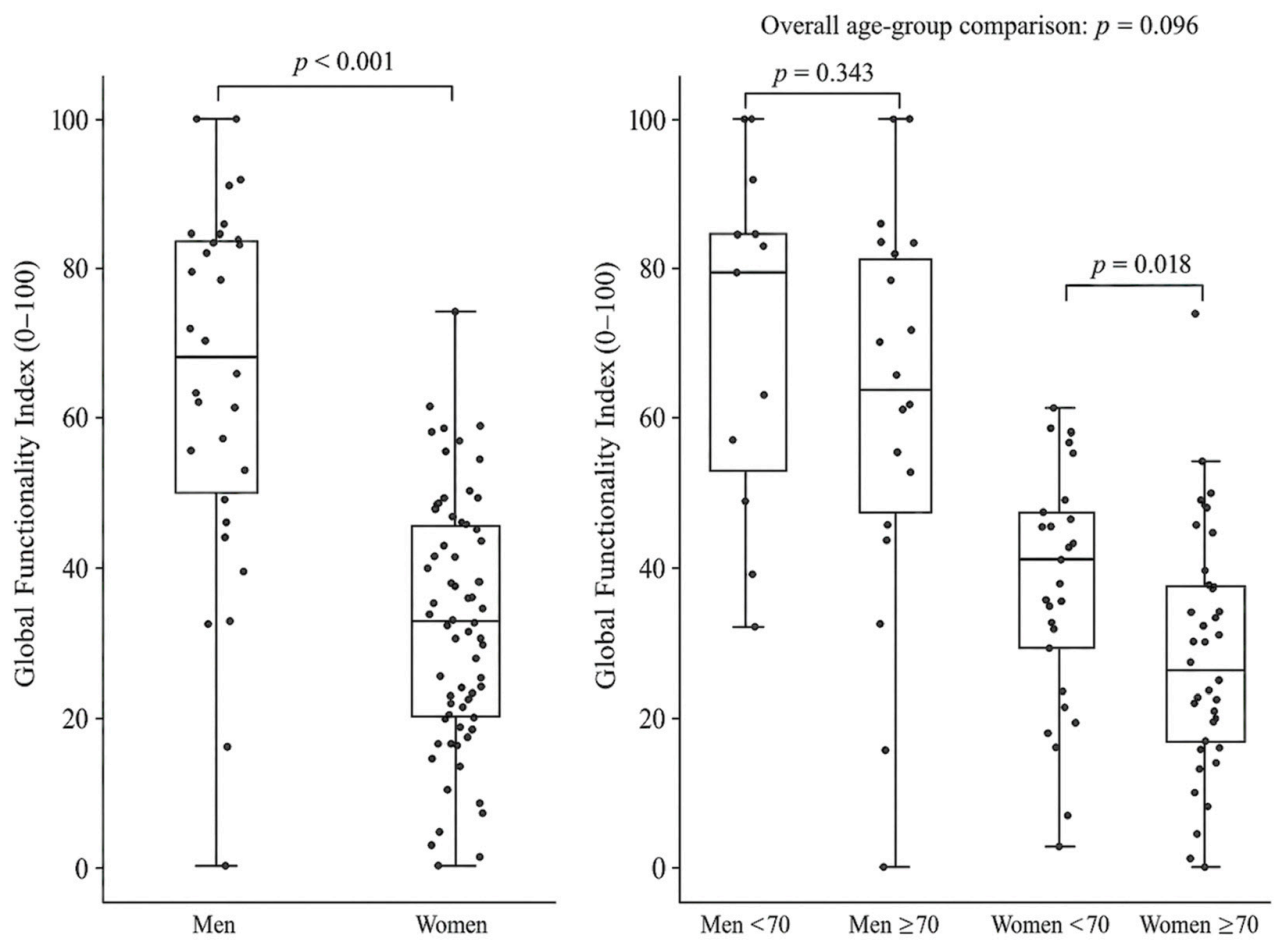
Distribution of Global Functionality Index values by sex and age group. Individual data points are shown with boxplot overlays.

The GFI differed significantly by sex but not by age group (Figure 4). Men exhibited higher GFI values than women (*p* < 0.001), indicating a substantial sex-related difference in functional capacity. Mean GFI values were 62.09 ± 23.92 in men and 31.63 ± 16.22 in women. The distribution of GFI scores stratified by sex is shown in Supplementary Figure S2. In contrast, the categorical comparison between participants younger than 70 years and those aged 70 years and older did not reach statistical significance for the overall GFI (*p* = 0.096). The sensitivity analysis based on sex-stratified standardization produced comparable results, indicating that the main findings were not dependent on pooled standardization of the GFI components.

The stratified analysis revealed a sex-specific age-associated pattern in GFI values. While no significant differences were observed between age groups in men (*p* = 0.343), women aged 70 years and older showed significantly lower GFI values than women younger than 70 years (*p* = 0.018). Consequently, women aged ≥ 70 years represented the subgroup with the lowest functional performance, whereas no significant age-group difference was detected among men. In the continuous analysis, age was negatively associated with GFI in the unadjusted correlation (r ≈ −0.26, *p* < 0.05), indicating lower GFI values at older ages within this cross-sectional sample. This association is illustrated by sex in Supplementary Figure S3. However, after adjusting for sex, age remained independently associated with GFI in the multivariable model. In addition, partial correlation analyses adjusted for age and sex revealed a significant inverse association between GFI and BMI (r = −0.24, *p* = 0.017), suggesting that higher BMI was associated with lower functional capacity. No other variables showed significant associations in partial correlation analysis.

A multivariable linear regression model including demographic, clinical, and lifestyle variables identified sex and age as the main independent correlates of the GFI. Female sex was associated with lower GFI values (β = −40.23, *p* < 0.001), according to the coding used in the model, while increasing age was independently associated with lower GFI values (β = −1.03, *p* < 0.001). BMI showed a borderline negative association (β = −0.97, *p* = 0.066). In contrast, no significant associations were observed for blood pressure, health-related quality of life (SF-12), cognitive performance, falls, comorbidity burden, or physical activity level (Table 2).

Regression diagnostics did not indicate major violations of model assumptions. Residuals were approximately normally distributed (Shapiro–Wilk W = 0.990, *p* = 0.857), homoscedasticity was supported (Breusch–Pagan *p* = 0.717), and multicollinearity was low (maximum VIF = 1.50). No highly influential observations were detected based on Cook’s distance (maximum Cook’s distance = 0.078), and the maximum leverage value was 0.433.

### 3.5. Identification of Functional Profiles and Exploratory Low-GFI Classification

As an internal exploratory analysis, binary logistic regression was used to examine factors associated with belonging to the lowest quartile of the GFI distribution. This sample-specific threshold was used to identify the lowest-functioning subgroup within the present cohort and should not be interpreted as a clinically validated cut-off. Age was associated with belonging to the lowest quartile of the GFI distribution (*p* = 0.008), indicating that older participants were more likely to be classified within the lowest GFI quartile. BMI showed a borderline association (*p* = 0.065), suggesting a possible relationship between higher BMI and lower functionality, although this did not reach statistical significance. The three-cluster solution provided an interpretable exploratory distribution of participants into low-, moderate-, and high-functionality profiles (Table 3). Significant differences across clusters were observed for all functional variables, with large effect sizes for HS, walk ratio, walking speed, and 6MWT (η^2^ ranging from 0.38 to 0.54; *p* < 0.001), whereas 10-STST showed a smaller effect size (η^2^ = 0.06, *p* = 0.047).

**Figure 5:**
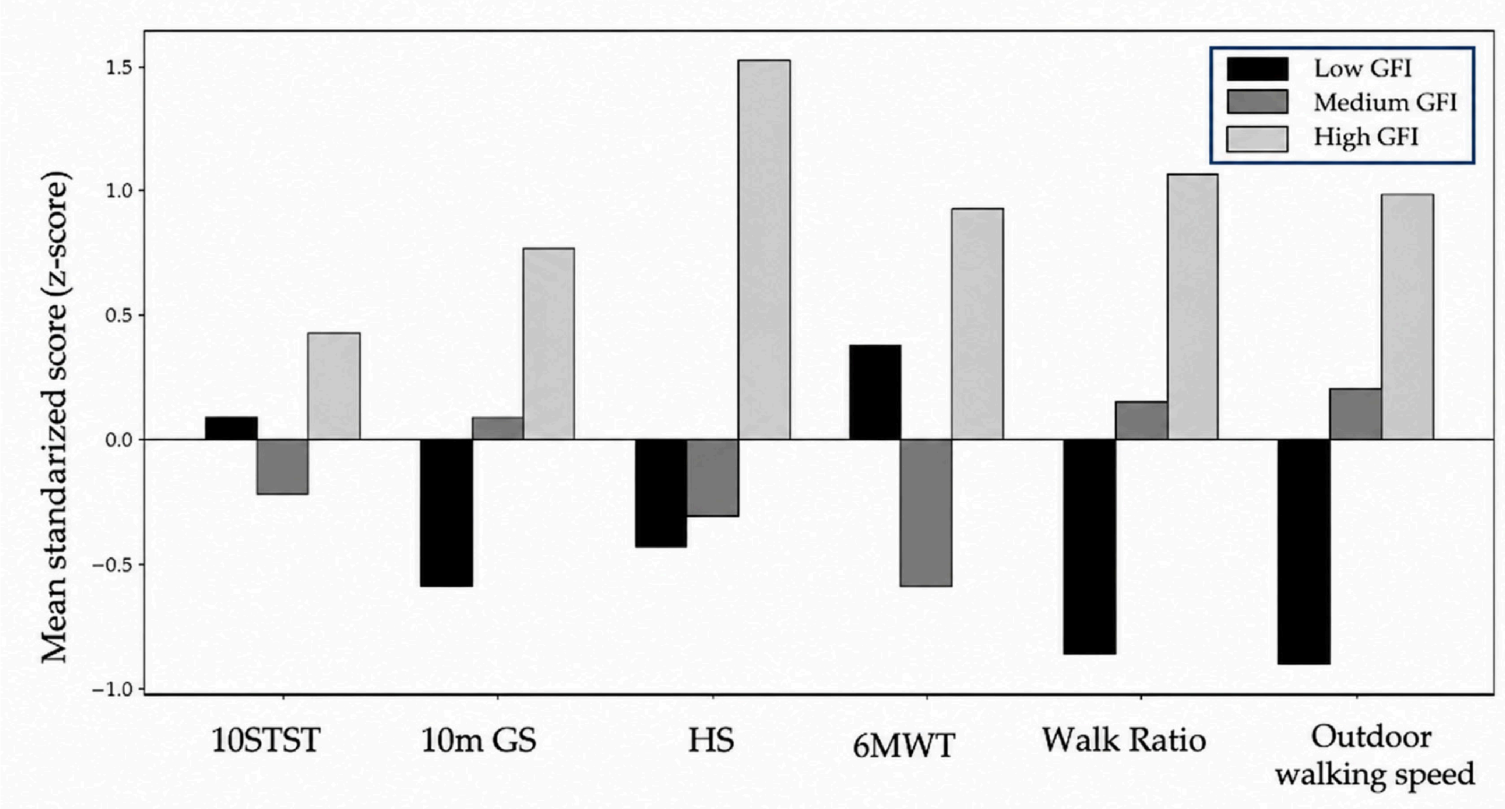
Cluster-based exploratory functional profiles according to the Global Functionality Index. Values represent standardized scores for each GFI component variable across the low-, moderate-, and high-functionality clusters.

The cluster analysis identified three distinct functional profiles (Figure 5). The high-GFI cluster showed the highest standardized scores across all components, whereas the low- and moderate-GFI clusters exhibited different patterns of functional limitations. The low-GFI cluster showed poorer performance particularly in the 10-STST and 6MWT, while the moderate-GFI cluster showed lower values for gait speed, walk ratio, and wearable-derived outdoor walking speed. These findings indicate heterogeneous rather than uniformly progressive functional profiles.

## 4. Discussion

The main objective of this study was to develop and internally evaluate a GFI in older adults and to examine the combined influence of age, sex, and anthropometric indicators on functional capacity. Overall, the findings suggest that the GFI may provide an interpretable derived index of functional capacity that integrates laboratory-based performance and wearable-derived outdoor gait metrics into a single continuous score. Rather than indicating a uniformly significant age-group effect, the results point to a sex-specific age-associated pattern, with lower functional performance particularly evident in women aged 70 years and older. The index identified three distinct functional profiles, underscoring the heterogeneity of functional aging.

The GFI addresses the need for functional assessment tools that move beyond single-domain or categorically scored instruments. Although established measures such as the Short Physical Performance Battery [11] and Fried’s frailty phenotype [12] remain central to geriatric assessment, the GFI provides a complementary continuous approach integrating strength, power, gait, endurance, and wearable-derived outdoor gait metrics. Compared with isolated gait speed or the Timed Up and Go, the GFI summarizes multiple functional domains rather than a single mobility task. Compared with the SPPB, it retains continuous information from strength, gait efficiency, endurance, and outdoor walking performance, which may help reduce ceiling effects in robust or pre-frail individuals [36]. Therefore, the proposed incremental contribution of the GFI is currently technological and methodological, through the integration of laboratory-based performance and wearable-derived outdoor walking data, rather than clinically demonstrated. Accordingly, the GFI should be considered a complementary research index rather than a replacement for established clinical tools. Direct benchmarking against the SPPB, Timed Up and Go, gait speed, and Frailty Index is required in future validation studies. In this sense, the present findings are consistent with the WHO Intrinsic Capacity framework, which conceptualizes functional health as multidimensional [37].

The exploratory PCA described a moderate internal structure of the selected functional variables and supported the interpretation of the GFI as a multidimensional rather than strictly unidimensional index. Two complementary domains were identified: a mobility-related component, mainly driven by gait efficiency, endurance, and wearable-derived outdoor walking performance, and a strength-related component, mainly reflecting neuromuscular capacity. These findings indicate that the selected variables capture related but not identical aspects of functional performance. Because the same variables were used to construct the GFI, PCA findings should be interpreted only as descriptive evidence of internal structure, not as validation of the index. Accordingly, PCA did not determine the final equal-weighted scoring procedure. Interpretations based on GFI component variables should be understood as descriptions of the index’s internal structure rather than as independent external validation. The strong agreement between the equal-weighted and PCA-weighted formulations further supports the stability of the main findings, although external validation is still required. Equal weighting was selected because it provides a transparent and easily reproducible scoring procedure, which is important for a newly derived index. However, this approach assumes that each component contributes similarly to global functional capacity. Expert-based weighting could introduce subjective decisions, whereas machine-learning-derived weighting would require larger samples and external outcome-based validation to avoid overfitting. PCA-derived weighting was explored as a sensitivity analysis, but it was not used as the primary scoring method because it would make the index more sample-dependent and less clinically interpretable. Although separate domain-specific sub-scores may be informative, they were not defined in the present derivation study and should be examined in future validation studies.

Age and sex were the most consistent correlates of functional capacity across analyses. Men showed higher GFI values than women, in line with previous reports of greater muscle strength and physical performance in men across the lifespan [7,38]. Because the primary GFI was based on pooled standardization, sex differences could theoretically be influenced by the reference distribution used for z-score calculation. However, the sex-stratified sensitivity analysis produced comparable results, suggesting that the observed sex-related pattern was not solely driven by the pooled normalization procedure. Age was inversely associated with GFI when treated as a continuous variable in crude and adjusted models. However, the categorical comparison between participants younger than 70 years and those aged 70 years and older did not show a significant overall difference in GFI. This suggests that the relationship between age and functional performance may be better captured by continuous age-associated variation than by this binary age-group classification. This interpretation is reinforced by the moderate association between GFI and age and is consistent with the concepts of biological aging and functional reserve, according to which aging trajectories are heterogeneous and only partially reflected by chronological age [39,40].

A relevant finding was that women aged ≥ 70 years showed lower GFI values than women younger than 70 years, whereas no significant age-group difference was detected among men. This pattern was most evident in strength-related domains and may reflect, at least partly, postmenopausal hormonal changes, including estrogen decline [41], together with lower peak muscle mass attained earlier in life [42,43]. By contrast, endurance-related differences were less pronounced. Although these mechanisms were not directly assessed, the results suggest that neuromuscular function may be relevant to sex-specific differences in functional performance in later life. More broadly, the findings are consistent with the morbidity–mortality paradox, whereby women live longer but often experience a greater burden of disability in later life [44]. However, the absence of a significant age-group difference in men should be interpreted cautiously given the small male subgroup size.

The association between BMI and GFI was modest. BMI was significantly associated with GFI in partial correlations, but its effect was attenuated in the multivariable linear model and remained only borderline in the exploratory logistic regression. Therefore, BMI should be interpreted only as a general anthropometric indicator rather than as a direct measure of adiposity or body composition. Direct measures of fat mass, lean mass, and muscle quality would be required to examine mechanisms related to sarcopenic obesity or body composition more specifically [45].

An additional finding was the limited contribution of clinical, cognitive, and self-reported health variables to objectively measured functional capacity. Blood pressure, health-related quality of life, cognitive performance, comorbidity burden, falls, and physical activity level were not independently associated with GFI in the multivariable model. These results suggest that performance-based function may represent a related but distinct physiological construct that is not fully captured by subjective health perception or conventional clinical indicators. From a geriatric perspective, this supports the added value of objective performance-based measures, particularly when the goal is to detect early vulnerability not yet evident in self-reported health status.

Cluster analysis further highlighted the heterogeneity of functional aging by identifying three distinct functional profiles. The differences observed across clusters illustrate the internal separation of functional profiles within this cohort; because the same variables were used to construct the GFI and define the clusters, this analysis does not constitute independent validation of the index. These profiles were functionally interpretable but should not be equated with clinically defined states of robustness, pre-frailty, or frailty described in gerontological research [12,46]. Building upon these profiles, the exploratory logistic regression showed that age was associated with belonging to the lowest quartile of the GFI distribution, whereas BMI contributed only marginally. This lowest-quartile threshold was sample-dependent and should not be interpreted as a clinical or diagnostic cut-off. Taken together, these results suggest that the GFI may help describe intermediate and low-functioning profiles within the present sample. However, its clinical usefulness will depend on whether it predicts meaningful longitudinal outcomes, including falls, disability, hospitalization, institutionalization, and mortality [47]. Therefore, prospective studies are required before the GFI can be considered a risk-prediction or decision-making tool.

### 4.1. Strengths and Limitations

Several limitations should be considered when interpreting these findings. First, the cross-sectional design precludes causal or temporal inferences; therefore, the regression results represent associations within the present cohort and do not allow the GFI to be interpreted as a predictor of future functional decline. Second, despite adequate statistical power, the sample’s characteristics—recruitment from senior associations in southern Spain, sex imbalance, urban predominance, and independently ambulatory status—limit generalizability to frailer, rural, institutionalized, clinically impaired, or internationally diverse older adults. Third, BMI served only as a general indicator and cannot differentiate fat mass, lean mass, or muscle quality. An additional limitation is that equal weighting assumes comparable contributions from all six components and may not reflect their differential clinical relevance or partial overlap. Fourth, the cluster solution was exploratory, and formal validation metrics such as silhouette coefficient, Davies–Bouldin index, or Calinski–Harabasz index were not applied. Additionally, the GFI is not a fall-risk-specific index; it omits fall-specific measures (balance, postural sway, gait variability, obstacle negotiation, dual-task gait, and prospective fall incidence), although future studies should examine whether their inclusion improves the prediction of slips, trips, or falls. Finally, although Garmin devices have previously been used to assess mobility and walking-related outcomes in older adults, validation evidence for the specific Garmin Forerunner 635/HRM-Pro configuration used in this study remains limited. Moreover, the use of a single outdoor assessment prevents evaluation of test–retest reproducibility and does not allow the resulting measurements to be considered representative of habitual daily life walking. Therefore, wearable-derived walking speed should be interpreted cautiously, as it may be affected by GPS signal variability, terrain, weather conditions, wrist movement artifacts, and device firmware or algorithm differences.

Importantly, because the GFI was developed and evaluated in a single cohort, the present findings should be interpreted as derivation and internal evaluation only. The analyses describe the internal structure and interpretability of the index, but do not establish construct, external, or predictive validity. Internal consistency was not considered a validation criterion, because the GFI was designed as a multidimensional composite index rather than as a set of interchangeable items. Future studies should examine test–retest reliability, compare the GFI with established functional tools, validate it in independent cohorts, and assess its predictive value for outcomes such as falls, disability, hospitalization, institutionalization, and mortality.

Despite these limitations, the present study has several notable strengths. First, it addresses a relevant topic in aging research by integrating laboratory-based physical performance tests with wearable-derived outdoor gait data obtained during a standardized walking task. Second, the multimodal assessment approach captures complementary neuromuscular, gait, endurance, and outdoor locomotor performance within a single analytical framework. Finally, translating continuous functional data into distinct profiles may provide a useful research framework for describing functional heterogeneity in older adults and for guiding future validation studies.

### 4.2. Practical Applications

From a practical perspective, the GFI may provide a useful research framework for summarizing multiple dimensions of functional performance in older adults. By integrating laboratory-based physical tests with wearable-derived outdoor gait data, the index offers an interpretable summary of functional performance and may help describe different functional profiles. The GFI score should be interpreted as a relative 0–100 summary of global functional capacity within the present sample, with higher values indicating better overall performance. For example, a score of 55 indicates that the participant’s composite performance lies 55% of the distance between the lowest and highest raw GFI values observed in this derivation sample; it does not represent a percentile rank or a validated clinical threshold. Accordingly, tentative categories such as low, moderate, or high GFI should not be assigned clinical meaning until reference values and externally validated cut-offs are established. In applied settings, the GFI could be calculated by collecting the six component measures and applying the standardized scoring procedure described in the Section 2.4 Statistical Analysis and Figure 2. The GFI may complement established geriatric assessment tools by providing a continuous summary of performance across several domains and by identifying component tests that may require closer examination; however, it should not replace validated instruments used for clinical screening, diagnosis, risk stratification, or decision-making. Its interpretation should remain descriptive at this stage, as external validation, reference values, clinically validated cut-offs, and prospective evidence in relation to relevant health outcomes are still required before using the GFI as a clinical screening, risk prediction, longitudinal monitoring, or decision-making tool.

## 5. Conclusions

In conclusion, the GFI represents a derived multidimensional index of functional capacity in older adults, integrating key domains of strength, mobility, endurance, gait efficiency, and wearable-derived outdoor walking performance. The index showed an interpretable internal structure and described functional profiles within the present sample, supporting its preliminary interpretation as a derivation-stage research summary. It also captured relevant demographic patterns, with higher functional scores in men and lower GFI values particularly evident in women aged 70 years and older. However, these findings should be interpreted as preliminary and limited to internal evaluation. Future studies should externally validate the GFI in larger and more diverse cohorts, assess its test–retest reliability, and examine its predictive value for relevant outcomes such as disability, falls, hospitalization, and functional decline.

## Figures and Tables

**Table 1:** Comparison by sex and age group.

Variable	Total n = 110	Men n = 34	Women n = 76	<70 Years n = 48	≥70 Years n = 62	*p*-Value (Sex)	*p*-Value (Age)	Cohen’s d (Sex)	Cohen’s d (Age)
BMI (kg/m^2^)	27.12 (3.92)	27.01 (3.33)	27.17 (4.19)	27.26 (4.43)	27.00 (3.51)	0.829	0.735	−0.041	0.070
WC (cm)	108.90 (22.05)	114.20 (20.53)	106.42 (22.43)	114.45 (25.57)	104.60 (17.94)	0.077	**0.026**	0.365	0.465
Systolic BP (mmHg)	130.4 (19.11)	139.57 (16.81)	126.50 (18.83)	130.43 (19.37)	130.38 (19.12)	**0.004**	0.990	0.720	0.003
Diastolic BP (mmHg)	81.97 (9.27)	85.92 (9.97)	80.00 (8.32)	83.14 (7.89)	80.95 (10.32)	**0.014**	0.301	0.670	0.243
10-STST (s)	17.58 (4.10)	16.26 (3.43)	18.18 (4.25)	16.85 (4.17)	18.15 (3.97)	**0.014**	0.102	−0.478	−0.320
10 m gait speed (m/s)	1.73 (0.24)	1.85 (0.28)	1.67 (0.19)	1.78 (0.26)	1.68 (0.21)	**<0.001**	0.032	0.840	0.430
HS (kg)	22.62 (6.11)	28.50 (6.03)	19.91 (3.84)	23.03 (6.18)	22.29 (6.09)	**<0.001**	0.528	1.859	0.121
6MWT (m)	606.93 (85.57)	633.87 (95.37)	595.33 (78.88)	602.14 (86.15)	610.23 (85.72)	0.054	0.640	0.467	−0.094
SF-12 MCS	50.80 (9.41)	50.65 (10.40)	50.87 (8.99)	51.89 (7.54)	49.81 (10.82)	0.925	0.317	−0.023	0.228
SF-12 PCS	49.81 (6.97)	50.08 (7.37)	49.68 (6.84)	49.69 (5.53)	49.91 (8.13)	0.819	0.886	0.060	−0.031
TYM	46.65 (4.80)	45.85 (4.45)	47.04 (4.96)	46.24 (5.17)	47.02 (4.48)	0.285	0.472	−0.250	−0.163
Charlson Index	1.90 (1.36)	2.31 (1.16)	1.70 (1.41)	2.26 (1.33)	1.57 (1.31)	**0.047**	**0.022**	0.456	0.523
Step length (m)	0.69 (0.06)	0.72 (0.06)	0.67 (0.05)	0.70 (0.04)	0.68 (0.07)	**<0.001**	**0.032**	0.939	0.390
Cadence (spm)	123.38 (6.24)	121.85 (5.95)	124.09 (6.28)	124.00 (6.18)	122.89 (6.29)	0.075	0.352	−0.362	0.180
Walk ratio	0.33 (0.04)	0.36 (0.03)	0.32 (0.03)	0.34 (0.03)	0.33 (0.04)	**<0.001**	0.199	1.333	0.248
Outdoor walking speed (m/s)	1.42 (0.14)	1.47 (0.14)	1.39 (0.13)	1.45 (0.12)	1.39 (0.15)	**0.004**	**0.030**	0.640	0.416

BMI: Body mass index; 10-STST: 10-repetition Sit-to-Stand-Test; HS: Handgrip strength; PCS: Physical component summary; MCS: Mental component summary; WC: Waist circumference; Spm: Steps per minute; TYM: Test your memory; Significant *p*-values are shown in bold.

**Table 2:** Independent associations with the Global Functionality Index.

Variable	Beta	95% CI	*p*-Value
const	174.76	103.17 to 246.35	<0.001
Age	−1.03	−1.58 to −0.48	<0.001
Sex	−40.23	−49.64 to −30.82	<0.001
BMI	−0.97	−2.00 to 0.06	0.066
SF12 Physical	0.14	−0.42 to 0.70	0.624
SF12 Mental	0.01	−0.40 to 0.41	0.962
TYM	−0.40	−1.23 to 0.44	0.344
Falls	1.03	−3.82 to 5.87	0.673
Charlson Index	0.86	−2.37 to 4.09	0.595
IPAQ	0.18	−6.21 to 6.56	0.956

BMI: Body mass index; TYM: Test your memory; IPAQ: International Physical Activity Questionnaire; Sex was coded as 0 = men and 1 = women.

**Table 3:** Comparison of functional performance variables across low-, moderate-, and high-GFI profiles.

Variable	Low GFI	Medium GFI	High GFI	*p*-Value	Effect Size (η^2^)
10-STST (s)	18.49 (4.43)	17.21 (3.90)	15.80 (3.40)	0.047	0.06
10 m speed (m/s)	1.75 (0.20)	1.59 (0.20)	1.91 (0.22)	<0.001	0.22
HS (kg)	20.59 (3.19)	19.83 (4.50)	31.73 (5.72)	<0.001	0.54
6MWT (m)	555.28 (67.86)	638.28 (67.31)	685.00 (72.53)	<0.001	0.38
Walk Ratio	0.34 (0.02)	0.30 (0.02)	0.37 (0.03)	<0.001	0.46
Walking speed (m/s)	1.44 (0.10)	1.29 (0.10)	1.54 (0.11)	<0.001	0.46

GFI: Global Functionality Index; 6MWT: 6 min walk test; HS: handgrip strength; 10-STST: 10-repetition sit-to-stand test.

## Data Availability

The data that support the findings of this study are available from the corresponding author upon reasonable request. To facilitate reproducibility, a supplementary Python script illustrating the calculation of the GFI and the main statistical procedures is provided as Supplementary File S1.

## Supplementary Materials

The following supporting information can be downloaded at: https://www.mdpi.com/article/10.3390/s26154722/s1, Figure S1: Sex-specific correlation heatmaps of the six component variables included in the Global Functionality Index. Figure S2: Distribution of Global Functionality Index scores stratified by sex. Figure S3: Scatterplot of Global Functionality Index values against age with separate regression lines for men and women. Table S1: Sociodemographic characteristics of participants stratified by sex and age group. Table S2: Principal component analysis of the six variables included in the Global Functionality Index. Supplementary File S1: Python script for GFI.

## Abbreviations

The following abbreviations are used in this manuscript:

6MWT	6 min walk test
BMI	Body Mass Index
CCI	Charlson Comorbidity Index
GFI	Global Functionality Index
GS	Gait speed
HS	Handgrip strength
IPAQ	International Physical Activity Questionnaire
MCS	Mental Component Summary
PA	Physical Activity
PCA	Principal Component Analysis
PCS	Physical Component Summary
SPPB	Short Physical Performance Battery
10-STST	10-repetitions sit-to-stand test
TYM	Test Your Memory
WC	Waist circumference
WHO	World Health Organization

## References

[1] World Health Organization (2016). Multisectoral Action for a Life Course Approach to Healthy Ageing: Draft Global Strategy and Plan of Action on Ageing and Health; Sixty-Ninth World Health Assembly A69/17.

[2] Rudnicka E., Napierała P., Podfigurna A., Męczekalski B., Smolarczyk R., Grymowicz M. (2020). The World Health Organization (WHO) approach to healthy ageing. Maturitas.

[3] Cesari M., De Carvalho I.A., Thiyagarajan J.A., Cooper C., Martin F.C., Reginster J.-Y., Vellas B., Beard J.R. (2018). Evidence for the domains supporting the construct of intrinsic capacity. J. Gerontol..

[4] Studenski S., Perera S., Patel K., Rosano C., Faulkner K., Inzitari M., Brach J., Chandler J., Cawthon P., Connor E.B. (2011). Gait speed and survival in older adults. JAMA.

[5] Middleton A., Fritz S.L., Lusardi M. (2015). Walking speed: The functional vital sign. J. Aging Phys. Act..

[6] van Kan G.A., Rolland Y., Andrieu S., Bauer J., Beauchet O., Bonnefoy M., Cesari M., Donini L.M., Gillette-Guyonnet S., Inzitari M. (2009). Gait speed at usual pace as a predictor of adverse outcomes in community-dwelling older people: An International Academy on Nutrition and Aging (IANA) Task Force. J. Nutr. Health Aging.

[7] Bohannon R.W. (2019). Grip strength: An indispensable biomarker for older adults. Clin. Interv. Aging.

[8] Benavent-Caballer V., Lisón J.F., Rosado-Calatayud P., Amer-Cuenca J.J., Segura-Ortí E. (2015). Factors associated with the 6-minute walk test in nursing home residents and community-dwelling older adults. J. Phys. Ther. Sci..

[9] Yee X.S., Ng Y.S., Allen J.C., Latib A., Tay E.L., Abu Bakar H.M., Ho C.Y.J., Koh W.C.C., Kwek H.H.T., Tay L. (2021). Performance on sit-to-stand tests in relation to measures of functional fitness and sarcopenia diagnosis in community-dwelling older adults. Eur. Rev. Aging Phys. Act..

[10] Patrizio E., Calvani R., Marzetti E., Cesari M. (2021). Physical functional assessment in older adults. J. Frailty Aging.

[11] Guralnik J.M., Simonsick E.M., Ferrucci L., Glynn R.J., Berkman L.F., Blazer D.G., Scherr P.A., Wallace R.B. (1994). A short physical performance battery assessing lower extremity function: Association with self-reported disability and prediction of mortality and nursing home admission. J. Gerontol..

[12] Fried L.P., Tangen C.M., Walston J., Newman A.B., Hirsch C., Gottdiener J., Seeman T., Tracy R., Kop W.J., Burke G. (2001). Frailty in older adults: Evidence for a phenotype. J. Gerontol..

[13] Rikli R.E., Jones C.J. (1999). Development and validation of a functional fitness test for community-residing older adults. J. Aging Phys. Act..

[14] Rockwood K., Mitnitski A. (2007). Frailty in relation to the accumulation of deficits. J. Gerontol..

[15] Kameniar K., MacKintosh S., Van Kessel G., Kumar S. (2024). The psychometric properties of the Short Physical Performance Battery to assess physical performance in older adults: A systematic review. J. Geriatr. Phys. Ther..

[16] Pua Y.-H., Tay L., Lin S., Lim S.-Q., Clark R.A., Thumboo J., Tay E.-L., Mah S.-M., Wang M.-X., Chew S.J.-Y. (2026). Validity and utility of a modified Short Physical Performance Battery assessment and scoring approach in middle-aged and older adults. J. Am. Med. Dir. Assoc..

[17] Alcazar J., Losa-Reyna J., Rodriguez-Lopez C., Alfaro-Acha A., Rodriguez-Mañas L., Ara I., García-García F.J., Alegre L.M. (2018). The sit-to-stand muscle power test: An easy, inexpensive and portable procedure to assess muscle power in older people. Exp. Gerontol..

[18] Beaudart C., Rolland Y., Cruz-Jentoft A.J., Bauer J.M., Sieber C.C., Cooper C., Al-Daghri N., Araujo de Carvalho I., Bautmans I., Bernabei R. (2019). Assessment of muscle function and physical performance in daily clinical practice: A position paper endorsed by the European Society for Clinical and Economic Aspects of Osteoporosis, Osteoarthritis and Musculoskeletal Diseases (ESCEO). Calcif. Tissue Int..

[19] Correa-de-Araujo R., Addison O., Miljkovic I., Goodpaster B.H., Bergman B.C., Clark R.V., Elena J.W., Esser K.A., Ferrucci L., Harris-Love M.O. (2020). Myosteatosis in the context of skeletal muscle function deficit: An interdisciplinary workshop at the National Institute on Aging. Front. Physiol..

[20] Donini L.M., Busetto L., Bischoff S.C., Cederholm T., Ballesteros-Pomar M.D., Batsis J.A., Bauer J.M., Boirie Y., Cruz-Jentoft A.J., Dicker D. (2022). Definition and diagnostic criteria for sarcopenic obesity: ESPEN and EASO consensus statement. Obes. Facts.

[21] Batsis J.A., Villareal D.T. (2018). Sarcopenic obesity in older adults: Aetiology, epidemiology and treatment strategies. Nat. Rev. Endocrinol..

[22] Minnetti M., Poggiogalle E., Frigerio F., Piciocchi C., Pierantozzi G., Di Vincenzo O., Pinto A., Gianfrilli D., Isidori A.M., Migliaccio S. (2026). Endocrinological aspects of sarcopenic obesity. Ann. Med..

[23] Giannouli E., Bock O., Mellone S., Zijlstra W. (2016). Mobility in old age: Capacity is not performance. BioMed Res. Int..

[24] Kawai H., Obuchi S., Watanabe Y., Hirano H., Fujiwara Y., Ihara K., Kim H., Kobayashi Y., Mochimaru M., Tsushima E. (2020). Association between daily living walking speed and walking speed in laboratory settings in healthy older adults. Int. J. Environ. Res. Public Health.

[25] de la Casa Pérez A., Latorre Román P.Á., Muñoz Jiménez M., Lucena Zurita M., Laredo Aguilera J.A., Párraga Montilla J.A., Cabrera Linares J.C. (2022). Is the Xiaomi Mi Band 4 an accuracy tool for measuring health-related parameters in adults and older people? An original validation study. Int. J. Environ. Res. Public Health.

[26] Gilgen-Ammann R., Schweizer T., Wyss T. (2020). Accuracy of distance recordings in eight positioning-enabled sport watches: Instrument validation study. JMIR Mhealth Uhealth.

[27] Megaritis D., Alcock L., Scott K., Hiden H., Cereatti A., Vogiatzis I., Del Din S. (2025). Real-world wrist-derived digital mobility outcomes in people with multiple long-term conditions: A comparison of algorithms. Bioengineering.

[28] Ferrucci L., Cooper R., Shardell M., Simonsick E.M., Schrack J.A., Kuh D. (2016). Age-related change in mobility: Perspectives from life course epidemiology and geroscience. J. Gerontol..

[29] Langeard A., Torre M.M., Temprado J.J. (2021). A dual-task paradigm using the Oral Trail Making Test while walking to study cognitive-motor interactions in older adults. Front. Aging Neurosci..

[30] Jenkinson C., Layte R., Jenkinson D., Lawrence K., Petersen S., Paice C., Stradling J. (1997). A shorter form health survey: Can the SF-12 replicate results from the SF-36 in longitudinal studies?. J. Public Health Med..

[31] Ojeda B., Salazar A., Dueñas M., Failde I. (2012). Traducción y adaptación al castellano del cuestionario de detección de trastorno cognitivo leve “Test Your Memory” (TYM). Med. Clin..

[32] Charlson M.E., Pompei P., Ales K.L., MacKenzie C.R. (1987). A new method of classifying prognostic comorbidity in longitudinal studies: Development and validation. J. Chronic Dis..

[33] Rubio Castañeda F.J., Tomás Aznar C., Muro Baquero C. (2017). Medición de la actividad física en personas mayores de 65 años mediante el IPAQ-E: Validez de contenido, fiabilidad y factores asociados. Rev. Esp. Salud Publica.

[34] Yanagawa N., Shimomitsu T., Kawanishi M., Fukunaga T., Kanehisa H. (2017). Relationship between performances of 10-time-repeated sit-to-stand and maximal walking tests in non-disabled older women. J. Physiol. Anthropol..

[35] Cabrera-Linares J.C., Martínez Salazar C., Párraga Montilla J.A., Latorre Román P.Á. (2025). Validation of Garmin HRM-Pro for assessment of spatiotemporal parameters during treadmill running: Agreement with three motion analysis systems. Sensors.

[36] Bhasin S., Travison T.G., Manini T.M., Patel S., Pencina K.M., Fielding R.A., Magaziner J.M., Newman A.B., Kiel D.P., Cooper C. (2020). Sarcopenia definition: The position statements of the Sarcopenia Definition and Outcomes Consortium. J. Am. Geriatr. Soc..

[37] López-Ortiz S., Lista S., Peñín-Grandes S., Pinto-Fraga J., Valenzuela P.L., Nisticò R., Emanuele E., Lucia A., Santos-Lozano A. (2022). Defining and assessing intrinsic capacity in older people: A systematic review and a proposed scoring system. Ageing Res. Rev..

[38] Alcazar J., Rodriguez-Lopez C., Delecluse C., Thomis M., Van Roie E. (2023). Ten-year longitudinal changes in muscle power, force, and velocity in young, middle-aged, and older adults. J. Cachexia Sarcopenia Muscle.

[39] López-Otín C., Blasco M.A., Partridge L., Serrano M., Kroemer G. (2023). Hallmarks of aging: An expanding universe. Cell.

[40] Ferrucci L., Gonzalez-Freire M., Fabbri E., Simonsick E., Tanaka T., Moore Z., Salimi S., Sierra F., de Cabo R. (2020). Measuring biological aging in humans: A quest. Aging Cell.

[41] Juppi H.K., Sipilä S., Fachada V., Hyvärinen M., Cronin N., Aukee P., Karppinen J.E., Selänne H., Kujala U.M., Kovanen V. (2022). Total and regional body adiposity increases during menopause—Evidence from a follow-up study. Aging Cell.

[42] Janssen I., Heymsfield S.B., Wang Z., Ross R. (2000). Skeletal muscle mass and distribution in 468 men and women aged 18–88 yr. J. Appl. Physiol..

[43] Dent E., Morley J.E., Cruz-Jentoft A.J., Arai H., Kritchevsky S.B., Guralnik J., Bauer J.M., Pahor M., Clark B.C., Cesari M. (2018). International clinical practice guidelines for sarcopenia (ICFSR): Screening, diagnosis and management. J. Nutr. Health Aging.

[44] Gordon E.H., Hubbard R.E. (2020). Differences in frailty in older men and women. Med. J. Aust..

[45] Cruz-Jentoft A.J., Bahat G., Bauer J., Boirie Y., Bruyère O., Cederholm T., Cooper C., Landi F., Rolland Y., Sayer A.A. (2019). Sarcopenia: Revised European consensus on definition and diagnosis. Age Ageing.

[46] Belfiori M., Salis F., Puxeddu B., Mulas M., Puligheddu M., Mandas A. (2026). Data-driven frailty and reserve phenotypes in older outpatients: A cluster analysis of complementary geriatric assessment. Front. Aging.

[47] Sánchez-Sánchez J.L., Lu W.-H., Gallardo-Gómez D., del Pozo Cruz B., de Souto Barreto P., Lucia A., Valenzuela P.L. (2024). Association of intrinsic capacity with functional decline and mortality in older adults: A systematic review and meta-analysis of longitudinal studies. Lancet Healthy Longev..

