# Supplementary material for: Development of a Global Functionality Index Integrating Physical Performance and Wearable-Derived Outdoor Walking Metrics in Older Adults
Source: Sensors (Basel). 2026 Jul 25;26(15):4722. doi: 10.3390/s26154722 (PMC13469237; doi:10.3390/s26154722)
Supplement: Supplementary file 1 [file sensors-26-04722-s001.zip › Supplementary Figure S3.pdf]

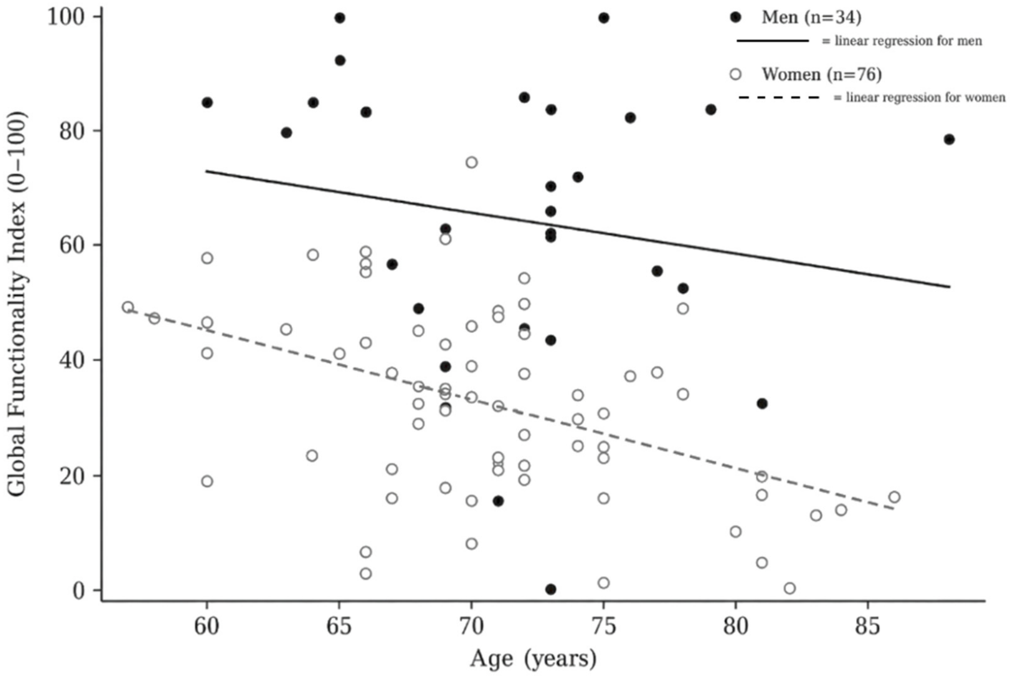

Supplementary Figure S3. Scatterplot of Global Functionality Index values against age with separate regression lines for men and women.
