# Supplementary material for: Development of a Global Functionality Index Integrating Physical Performance and Wearable-Derived Outdoor Walking Metrics in Older Adults
Source: Sensors (Basel). 2026 Jul 25;26(15):4722. doi: 10.3390/s26154722 (PMC13469237; doi:10.3390/s26154722)
Supplement: Supplementary file 1 [file sensors-26-04722-s001.zip › Supplementary Table S1.pdf]

Supplementary Table S1. Sociodemographic characteristics of participants stratified by sex and age group.

Variable		Total (n=110)	Men (n=34)	Women (n=76)	p-value	<70 years (n=48)	≥70 years (n=62)	p-value
Age, years (Mean ± SD)		70.56 ± 6.32	71.03 ± 5.70	70.35 ± 6.61	0.728	65.29 ± 3.74	74.95 ± 4.37	<0.001
Educational level n (%)	Low	25 (22.7)	3 (8.8)	22 (28.9)	0.032	13 (26.5)	11 (18.6)	0.123
	Medium	40 (36.4)	17 (50.0)	23 (30.3)		21 (42.9)	18 (30.5)	
	High	45 (40.9)	14 (41.2)	31 (40.8)		15 (30.6)	30 (50.8)	
Employment status n (%)	Retired	99 (90.0)	33 (97.1)	66 (86.8)	0.229	44 (89.8)	53 (89.8)	0.631
	Active/working	10 (9.1)	1 (2.9)	9 (11.8)		5 (10.2)	5 (8.5)	
	Other	1 (0.9)	0 (0.0)	1 (1.3)		0 (0.0)	1 (1.7)	
Previous/current occupation, n (%)	No	52 (47.3)	15 (44.1)	37 (48.7)	0.526	20 (40.8)	31 (52.5)	0.631
	Yes	58 (52.7)	19 (55.9)	39 (51.3)		29 (59.2)	28 (47.5)	
Marital status n (%)	Married	73 (66.4)	25 (73.5)	48 (63.2)	0.156	31 (63.3)	41 (69.5)	0.274
	Single	6 (5.5)	0 (0.0)	6 (7.9)		4 (8.2)	2 (3.4)	
	Widowed	23 (20.9)	5 (14.7)	18 (23.7)		9 (18.4)	14 (23.7)	
	Divorced	8 (7.3)	4 (11.8)	4 (5.3)		5 (10.2)	2 (3.4)	
Living alone n (%)	Yes	72 (65.5)	21 (61.8)	51 (67.1)	0.595	34 (69.4)	37 (62.7)	0.377
	No	38 (34.5)	13 (38.2)	25 (32.9)		15 (30.6)	22 (37.3)	
Residential area n (%)	Urban	108 (98.2)	34 (100.0)	74 (97.4)	1	48 (98.0)	58 (98.3)	1
	Rural	2 (1.8)	0 (0.0)	2 (2.6)		1 (2.0)	1 (1.7)	
Tobacco consumption n (%)	Non-smoker	65 (59.1)	20 (58.8)	45 (59.2)	0.786	27 (55.1)	36 (61.0)	0.131
	Ocasional smoker	11 (10.0)	4 (11.8)	7 (9.2)		8 (16.3)	3 (5.1)	
	Daily smoker	9 (8.2)	4 (11.8)	5 (6.6)		2 (4.1)	7 (11.9)	
	Ex-smoker	25 (22.7)	6 (17.6)	19 (25.0)		12 (24.5)	13 (22.0)	

Alcohol consumption n (%)	None	58 (52.7)	13 (38.2)	45 (59.2)	0.082	23 (46.9)	35 (59.3)	0.236
	Moderate	51 (46.4)	20 (58.8)	31 (40.8)		25 (51.0)	24 (40.7)	
	High	1 (0.9)	1 (2.9)	0 (0.0)		1 (2.0)	0 (0.0)	

Data are presented as n (%) except for age, which is presented as mean ± SD.
