# Supplementary material for: Development of a Global Functionality Index Integrating Physical Performance and Wearable-Derived Outdoor Walking Metrics in Older Adults
Source: Sensors (Basel). 2026 Jul 25;26(15):4722. doi: 10.3390/s26154722 (PMC13469237; doi:10.3390/s26154722)
Supplement: Supplementary file 1 [file sensors-26-04722-s001.zip › Supplementary Table S2.pdf]

Supplementary Table S2. Principal component analysis of the six variables included in the Global Functionality Index.

A. PCA summary

PCA summary	PC1	PC2	PC3	PC4	PC5	PC6
Eigenvalue	2.252	1.074	0.948	0.733	0.551	0.441
Explained variance (%)	37.53	17.91	15.81	12.21	9.19	7.35
Cumulative variance (%)	37.53	55.44	71.25	83.46	92.65	100.00

B. Component loadings and communalities

Variable	PC1 loading	PC2 loading	Communality
10-STST, inverted	0.260	0.889	0.858
10-m gait speed	0.706	0.207	0.541
HS	0.697	0.094	0.495
6MWT	0.347	0.073	0.125
Walk ratio	0.721	−0.424	0.700
Free-living walking speed	0.748	−0.217	0.607

Note. PCA was performed on standardized complete-case data for the six GFI component variables. The 10-STST was inverted before standardization so that higher values indicated better performance. Communalities refer to the variance explained by PC1 and PC2.
